# A Short Report on the Impact of Judo on Behaviors and Social Skills of Children With Autism Spectrum Disorder

**DOI:** 10.7759/cureus.41516

**Published:** 2023-07-07

**Authors:** Blake S Lockard, Marissa Dallara, Chasity O’Malley

**Affiliations:** 1 Medicine and Surgery, Nova Southeastern University Dr. Kiran C. Patel College of Allopathic Medicine, Davie, USA; 2 Allopathic Medicine, Nova Southeastern University Dr. Kiran C. Patel College of Allopathic Medicine, Davie, USA; 3 Medical Education, Wright State University Boonshoft School of Medicine, Dayton, USA

**Keywords:** quality of life, development, behavorial therapy, social behavior, empathy and social cognition, language and communication, judo, autism spectrum disorder (asd)

## Abstract

Introduction: Autism spectrum disorder (ASD) is a neurodevelopmental disorder characterized by communication difficulty and social deficits. The current treatment employs the use of psychosocial therapy and medication as well as alternative treatments. This is a pilot study that assessed whether participation in judo improved behavior and social skills in children with ASD.

Methods: Twenty-four students from the Riverside Youth Judo Club were included in the study, after obtaining consent from their parents to participate. Inclusion criteria included participation in judo classes for more than one month and diagnosis of ASD and/or a developmental disability. Parent(s) of the children signed a consent form, filled out a study questionnaire and completed the Social Skills Improvement System Social-Emotional Learning Edition, Parent Form (SSIS-SEL). Parents also had the opportunity to volunteer their child’s baseline SSIS-SEL assessment. SSIS-SEL data was taken for four participants and compared to the baseline.

Results: In the study questionnaire, 62.5% of parents agreed that their children demonstrated improvement across all six categories. The category with the greatest improvement was ‘Behavior at home’, while the category with the least improvement was ‘Eye contact’.

Conclusion: While the direct impact of judo on special needs children was difficult to assess due to variability in abilities and developmental milestones, we hope that improving awareness regarding the effectiveness of youth sports would impact the long-term quality of life for children with any developmental or mental disability and may help improve their social and behavioral skills in multiple environments.

## Introduction

Autism spectrum disorder (ASD) is a neurodevelopmental disorder that develops in a child due to a complex etiology involving both genetic and environmental components [1]. Fundamental issues of ASD involve deficits in social communication along with restricted interests and repetitive behaviors [2]. Children with ASD are often afflicted with other somatic and psychiatric comorbidities including attention deficit hyperactivity disorder (ADHD), anxiety, depressive disorders, epilepsy, intellectual disability (ID), sleep disorders, sight and hearing impairment/loss, and gastrointestinal syndromes [3].

One of the primary modalities of behavioral treatment for ASD is applied behavior analysis (ABA) that focuses on rewarding desired behaviors and ignoring inappropriate behaviors [4]. The practice of ABA can include one-on-one sessions, educating parents with step-by-step instructions, and providing the child with exposure to environmental stimuli.

Alternative treatment approaches include integration of self-management strategies into mainstream education [5]. Self-management allows children with ASD to regulate and monitor their behavior, while learning how to differentiate between appropriate and inappropriate behavior. Additionally, social groups at school can have a positive impact on child engagement and improve interaction with others [6]. Furthermore, martial arts have been explored as an alternative treatment approach to improve social skills, self-esteem, communication, and motor ability [7-10]. Martial arts can foster mindfulness and combat stress, which can be therapeutic for children with special needs [11]. The practice of judo can improve maladaptive behaviors in children with ASD including repetitive behaviors, emotional responses, and maladaptive speech [9,12]. Other notable benefits of judo for children with ASD are improvement in executive functions and the promotion of physical activity to limit sedentary behavior [12-13]. Judo may be therapeutic for children with ASD by creating a unique environment where they can communicate, follow directions, feel comfortable around others, manage behaviors after losing, and gain confidence through dedication and improvement.

The Riverside Police Foundation’s Youth Judo Club in Riverside, California, is dedicated to teaching the traditional art of Kodokan Judo to the largest group of developmentally disabled students in the United States. This study followed children who are members of the Riverside Police Foundation’s Youth Judo Club. The club teaches over 65 students with various mental and physical disabilities including ASD, tuberous sclerosis, and cerebral palsy, and also incorporates children without developmental disabilities into their classes.

Prior studies explored alternative interventions that allowed for the measurement of improvement in social skills in disabled children over the course of the intervention [9-10,14]. The goal of this pilot study was to evaluate children with ASD who participated in judo classes for more than a month to measure the impact of judo on improvement in behavior and social skills.

## Materials and methods

Participants and demographics

A total of 24 children participated in this pilot study, with 15 male students and 9 female students. Inclusion criteria included active membership at the Riverside Police Foundation’s Youth Judo Club, participation in judo classes for longer than one month and a diagnosis of ASD and/or developmental disability. Children over the age of 18 who were unable to make decisions due to intellectual disability were also included in the study. Social Skills Improvement System Social-Emotional Learning Edition (SSIS-SEL) data was collected from four of the participants; two of them were male and two were female (n=4). The average length of participation in the club was approximately two years, not inclusive of two participants who omitted the length of participation; ages ranged between 4 and 23 years.

Ethical approval

An Institutional Review Board (IRB) approval (2022-313-NSU) was obtained from the Nova Southeastern University prior to the start of data collection. Parent(s) or caregivers of children who met inclusion criteria gave consent to complete surveys on behalf of their children.

Study questionnaire

A questionnaire was created for this study to measure behavioral improvements after participation in judo. All 24 parents, who agreed to the study, completed the study questionnaire. Measurements of behavioral improvement included the following: ‘Behavior at home’, ‘Eye contact’, ‘Behavior at school’, ‘Ability to share’, ‘Social skills’, and ‘Performance in school’. The questionnaire employed the use of a 6-point Likert-type scale to measure the degree of agreement or disagreement with indicators ranging from ‘strongly disagree’ to ‘strongly agree’. A neutral indicator was not included in this scale in order to elicit valuable feedback from parents and caregivers and circumvent the issue of neutral questionnaire responses being chosen to bypass a purposeful thought process. Unique comments written by parents were analyzed qualitatively in addition to the survey results.

SSIS-SEL

Parents additionally filled out a Social Skills Improvement System Social-Emotional Learning Edition, Parent Form if their child had an initial SSIS-SEL assessment as a baseline measure. This survey measures five Social-Emotional Learning competencies: self-awareness, self-management, social awareness, relationship skills, responsible decision making and core skills. Children who had a baseline SSIS-SEL assessment were concurrently participating in ABA therapy. The baseline data for these four participants was obtained from their board-certified behavior analyst prior to ABA therapy. Baseline SSIS-SEL as well as four new SSIS-SEL assessments were collected that were administered to measure improvement after participation in judo. Means, standard deviations, and significance values were analyzed from the baseline and post-survey data.

## Results

Study questionnaire outcomes

Scores reported in Table 1 use -3 to -1 to represent varying levels of disagreement, with -3 corresponding to ‘strongly disagree’, -2 corresponding to ‘disagree’, and -1 corresponding to ‘slightly disagree’, while 1 to 3 represent varying levels of agreement, with 1 corresponding to ‘slightly agree’, 2 corresponding to ‘agree’, and 3 corresponding to ‘strongly agree’. The categories with the most profound difference were ‘Behavior at home’ (M±SD = 2.58±0.58), ‘Behavior at school’ (M±SD = 2.39±0.78), ‘Social skills’ (M±SD = 2.38±0.77), and ‘Performance in school’ (M±SD = 2.39±0.78). The least improvement was seen in ‘Eye contact’, but despite being the category with the least improvement, 87.5% of parents selected ‘slightly agree’ or better. ‘Eye contact’ (M±SD = 2.09±1.02) and ‘Ability to share’(M±SD = 2.14±1.32) had the most variability in responses and were the only two categories in which some parents selected either ‘slightly disagree’ or ‘disagree’ on the questionnaire. A total of 62.5% of parents agreed that their children demonstrated behavioral improvement through participation in the judo club across all behavioral categories from the study questionnaire; 8.33% of parents selected ‘slightly disagree’ or worse (‘disagree’ or ‘strongly disagree’) in at least one category and 29.1% of parents selected ‘N/A’ in at least one category.

**Table 1:** Results from all categories of the study questionnaire Results include means, standard deviations, and range and number of responses recorded for each section.

Measures	M	SD	Range	n
Age (years)	10.29	5.47	4–23	24
Length of participation (years)	1.95	1.93	0.08–7	22
Behavior at home	2.58	0.58	1–3	24
Eye contact	2.09	1.02	-1–3	22
Behavior at school	2.39	0.78	1–3	18
Ability to share	2.14	1.32	-2–3	22
Social skills	2.38	0.77	1–3	24
Performance in school	2.39	0.78	1–3	18

SSIS-SEL outcomes

The average length of participation in the club for the participants for whom SSIS-SEL data was collected was approximately nine months. These four children's averages improved across all competencies measured in the SSIS-SEL except for the core skills competency, which is represented in Table 2. The greatest improvement for these participants was in ‘Social awareness’ while the least improvement was seen in ‘Responsible decision making’. Baseline composite scores (M±SD = 76.5±28.16) were compared to post composite scores (M±SD = 80±21.21), but there was no significant difference (p=0.801). The widest variability in responses was in baseline measures of ‘Social awareness’ (M±SD = 80±31.46). The least variability in responses was in baseline measures of ‘Self-awareness’ (M±SD = 73.75±19.59). It is important to note that one of the four children from whom SSIS-SEL data was collected had declining scores after their baseline measurement for unknown reasons, which may have skewed the data by decreasing the average improvement from baseline for all four participants.

**Table 2:** Composite SSIS-SEL scores compared to the baseline for four children with ASD ASD, autism spectrum disorder; SSIS-SEL, Social Skills Improvement System Social-Emotional Learning Edition Means, standard deviations, and range are included for each category.

Measures	M	SD	Range
SEL composite (baseline)	76.50	28.16	47–107
SEL composite (post)	80.00	21.21	58–109
Self-awareness (baseline)	73.75	19.59	48–89
Self-awareness (post)	77.75	22.14	53–105
Self-management (baseline)	81.25	20.43	60–107
Self-management (post)	83.50	17.48	66–107
Social awareness (baseline)	80.00	31.46	53–119
Social awareness (post)	85.75	17.06	65–106
Relationship skills (baseline)	87.25	26.97	62–113
Relationship skills (post)	88.25	21.47	66–115
Responsible decision making (baseline)	82.50	20.87	56–102
Responsible decision making (post)	83.25	14.73	68–102
Core skills (baseline)	84.25	23.37	59–110
Core skills (post)	82.50	15.18	71–103

## Discussion

The results of this pilot study demonstrated that judo had a positive impact on the behaviors and social skills of children with ASD in multiple environments. Few parents reported negative responses in the ‘Eye contact’ and ‘Ability to share’ categories in the study questionnaire (Table 1). Parent responses on the SSIS-SEL demonstrated an average improvement of 5 points from the baseline, but these results were not statistically significant due to the small sample size of four children (Table 2). These findings were consistent with the study conducted by Morales et al., which demonstrated progress in social interaction, repetitive behaviors, and social communication over an eight-week intervention for children with ASD [9]. These findings vary from the study by Rivera et al., which reported improvement in social skills but no change in ASD-related behavior over an eight-week intervention [10]. Although parent-reported questionnaire responses may help to identify areas in which judo provides a benefit, an increased sample size for SSIS-SEL data can help to validate this improvement in behavior.

One unique aspect of this study is that participants with a wide range of developmental abilities were included, including non-verbal participants with ASD. This inclusion may provide further insights into the benefits that martial arts may have for children on all parts of the spectrum. The comment section of the questionnaire provided strong evidence that children demonstrated improvement in both behavior and social skills after participation in judo. Comments recorded included, “It has opened our eyes to some hope regarding physical activity”, “He learned how to play tag with other kids and before he would never acknowledge children”, “Judo helped my daughter calm herself and socialize with others”, “He loves to assist younger students”, and “He has social anxiety, so this has helped him a lot”. Many children with ASD have different developmental goals, but every improvement can help a child reach a new milestone and improve their quality of life. One of the goals of this study is to not only demonstrate that students with disabilities can improve regardless of their starting point, but to also recommend that families consider extracurricular activities as alternative or additional treatment.

One limitation of the study was the small sample size. A larger sample size would be beneficial in future studies to validate the effects that martial arts may have on behavior and social skills in children with ASD. In addition, the implementation of a validated survey such as the SSIS-SEL would yield more meaningful data that could affect new treatment options for children with ASD. This brings up another limitation of the study: the study questionnaire that was developed for the study was not a validated survey. Some of the difficulties that were found in creating a validated survey was the fact that the participants had participated in the judo club for an average of two years, with many participants either above or below this average, so measuring outcomes with different participation lengths of time proved difficult.

Another limitation was determining whether judo itself had an impact on the study results. This was difficult to determine because all four children for whom SSIS-SEL survey data was collected were currently receiving ABA therapy at home for a minimum of six months, from the previously recorded SSIS-SEL. ABA therapy allows a child to work on social and behavioral skills with an in-home therapist and involves collection of data to monitor progress in reaching developmental goals. Regardless of these possible confounding variables, observed improvement was reported across all children involved in judo. Another factor that was not recorded was consistency of participation; some students attend judo classes weekly while other students attend classes on a more infrequent basis. Future studies may collect this information for more validated results to distinguish improvement from participation in judo classes from participation in ABA therapy. Finally, a factor that may have contributed to overall improvement could have been that many of the instructors were trained in treating inappropriate behavior in children with ASD, so this may have been another possible confounding variable. Validation and understanding of the needs of children with ASD may help with their improvement in unique environments.

## Conclusions

Parents of children who are diagnosed with ASD may find it challenging to find activities for their children to participate in. Many of these children have different abilities and developmental milestones. We were able to explore parents’ perspectives on the effects of judo on their children in several categories including eye contact, social skills, behaviors at home, behaviors at school, ability to share, and performance in school. While the direct impact of judo on special needs children was difficult to assess due to variability in development, parent questionnaire responses and comments demonstrated improvements across all behavioral and social categories. The important conclusions of this pilot study are that participating in judo in the long term may help children with special needs improve their social and behavioral skills in multiple environments and improve their quality of life.
